# Effectiveness and safety of aerobic exercise for rheumatoid arthritis: a systematic review and meta-analysis of randomized controlled trials

**DOI:** 10.1186/s13102-022-00408-2

**Published:** 2022-02-05

**Authors:** Hui Ye, Heng Weng, Yue Xu, Lulu Wang, Qing Wang, Guihua Xu

**Affiliations:** School of Nursing, Nanjing University of Chinese Medicine, No. 138, Xianlin St., Box 064, Nanjing, 210023 People’s Republic of China

**Keywords:** Aerobic exercise, Rheumatoid arthritis, Function ability, Disease activity, Systematic review, Meta-analysis, Randomized controlled trials

## Abstract

**Background:**

Rheumatoid arthritis (RA) can cause severe physical impairment and a reduced quality of life, and there is limited evidence for any effective intervention. Aerobic exercise may be beneficial for improving symptoms. Therefore, the purpose of this meta-analysis was to evaluate the effectiveness and safety of aerobic exercise for rheumatoid arthritis patients.

**Methods:**

PubMed, The Cochrane Library, Web of Science, EMBASE, CNKI, WanFang Data and VIP databases were searched. Randomized controlled trials of the effectiveness and safety of aerobic exercise for rheumatoid arthritis were included. Risks of bias were assessed by two independent reviewers using the methods described in the RevMan 5.3, GRADEpro and the Cochrane Handbook. Meta-analyses were performed to investigate the effects of aerobic exercise on rheumatoid arthritis.

**Results:**

A total of 13 RCTs were included, including 967 rheumatoid arthritis patients. The Meta-analysis results showed that aerobic exercise can improve functional ability [MD = − 0.25, 95% CI (− 0.38, − 0.11), *P* = 0.0002], relieve pain [SMD = − 0.46, 95% CI (− 0.90, − 0.01), *P* = 0.04], increase aerobic capacity [MD = 2.41, 95% CI (1.36, 3.45), *P* < 0.00001] and improve the Sit to Stand test score[MD = 1.60, 95% CI (0.07, 3.13), *P* = 0.04] with statistically significant differences.

**Conclusion:**

Generally, aerobic exercise is beneficial and safe for RA patients and has a certain alleviating effect on the disease, such as functional ability improvement, pain relief and aerobic capacity increase. Limited by the quantity and quality of the included studies, future research with higher-quality studies needs to be conducted to verify the above conclusions.

*Trial registration*: PROPERO registration number: CRD42021242953.

## Introduction

Rheumatoid arthritis (RA) is a chronic and systemic inflammatory autoimmune disease that mainly involves the joints [1], with an estimated prevalence between 0.3 and 1%. It is more commonly diagnosed in women [2, 3]. The age-standardized prevalence and incidences of RA are increasing globally [4]. RA patients usually show painful symmetrical joint swelling, morning stiffness and limited functioning of the joints [5], leading to functional disability to varying degrees [6] and a decline in quality of life. The pathological process of RA involves abnormal activation and proliferation of macrophages and B and T lymphocytes, along with oxidative stress damage in human body, causing joint destruction, activating the immune system, and ultimately leading to a chronic inflammatory response [7, 8].

Currently, in RA treatment, priority is given to pharmacotherapy. Nonsteroidal anti-inflammatory drugs (NSAIDs) and disease-modifying antirheumatic drugs (DMARDs) are commonly used in clinical practice [9, 10]. The advent of biological therapeutics as an important segment of pharmacological treatment has been a major advance in the treatment of RA [11]. Medicine has achieved certain effects; however, there are disadvantages of adverse drug reactions, such as gastrointestinal adverse reactions, high cost and limited efficacy [12, 13], and nonpharmacological therapies are providing emerging support. Therefore, people have become increasingly aware of the importance of exploring other treatments for RA.

An increasing number of studies have suggested that increasing physical activity and exercise can improve symptoms and reduce the impact of systemic manifestations in RA [14, 15]. Physical activity is defined as any bodily movement produced by skeletal muscles that results in energy expenditure. Exercise is a subset of physical activity that is planned, structured, repetitive and has as a final or an intermediate objective the improvement or maintenance of physical fitness [16]. Exercise modalities, including aerobic exercise, resistance training and strength training, have been proven to be beneficial to the disease management of RA [17]. Therapeutic exercise can increase biomolecular suppression of arthritis through the suppression of inflammatory cytokine expression, consequently suppressing joint destruction [18]. Physical activity has been recommended to be a component of standard care for RA patients by The European League Against Rheumatism [19].

Aerobic exercise predominantly depends on the aerobic energy-generating process, including jogging, walking and cycling, and aims to increase peak oxygen consumption (VO_2_max) by increasing the heart rate to 50%–80% of the maximum heart rate [20]. An investigation found a significant correlation between aerobic exercise habits and the onset of RA, and good aerobic exercise habits can reduce the risk of RA [21]. Researchers have found that aerobic exercise can significantly reduce joint damage [22], reduce the risk of cardiovascular disease [23], relieve symptoms of fatigue [24], improve the muscle strength of RA patients [25] and improve their quality of life [26]. At the same time, aerobic exercise has the advantages of having few toxic side effects, has a low cost, and is generally acceptable to patients.

To summarize the latest evidence of an effect of an aerobic exercise program in rheumatoid arthritis and to obtain a better estimate of the mean effect, we performed the current meta-analysis. This study followed AMSTAR 2.0 [27] guidance for systematic meta-analyses, reported the data in accordance with the Preferred Reporting Items for Systematic Reviews and Meta-Analyses guidelines [28], provided evidence of the methodological and reporting quality supporting the intervention impact, and clarified the effects and safety of aerobic exercise for RA patients.

## Methods

### Protocol and registration

This systematic review has been registered in PROSPERO, 2021 (registration number: CRD42021242953). It was completed and structured according to the Preferred Reporting Items for Systematic Reviews and Meta-Analyses (PRISMA) guidelines.

### Search strategy

This systematic review and meta-analysis were performed in accordance with the PRISMA statement. The search was conducted by two reviewers, independent of each other. Randomized controlled trials (RCTs) and matched case studies were identified by searching PubMed, The Cochrane Library, Web of Science, EMBASE, and three Chinese electronic databases (China National Knowledge Infrastructure, Chinese VIP Information Database and WanFang Med Database) from 2000 until January 2022, using medical subject heading (MeSH) terms and all synonyms for “rheumatoid arthritis” in combination with the MeSH term and all synonyms for “aerobic exercise”. In addition, we manually searched the references of the included literature to supplement access to all relevant literature. The search terms and strategy for PubMed are provided in Appendix 1. References to all identified publications were entered into reference managing software (Endnote version X9.3.3).

### Selection criteria

Studies that met the following eligibility criteria were included: (1) designed as an RCT or matched case study; (2) published in English or Chinese; (3) patients, irrespective of sex or age, who were already clinically diagnosed with rheumatoid arthritis as defined by the American College of Rheumatology criteria or European League Against Rheumatism 2010 classification criteria [29]; (4) aerobic exercise interventions performed at 50–90% of the maximal heart rate, including walking, cycling, jogging, etc.; (5) presence of a control group, including but not limited to usual care, health education, or other exercise methods. All other treatments were required to be consistent between the experimental group and the control group. (6) Predefined inclusion criteria were data on functional ability, disease activity, joint counts, inflammatory markers, pain and aerobic capacity. As part of a sensitive search strategy, no restriction was initially made for specific outcomes. The exclusion criteria mainly were studies that were repeated publications, the full text was not available, the original research data were missing or the necessary data could not be extracted.

### Data extraction, endpoints

Two researchers screened the literature independently, extracted the data and cross-checked them. Disagreements were resolved through discussion or negotiation with a third reviewer. Titles and abstracts were read first to exclude any obviously irrelevant literature. Next, the full texts of the remaining articles were reviewed for final inclusion. The data extraction content included ① basic information of the included research: title, author, year, etc.; ② baseline characteristics and intervention measures; ③ outcome indicators [30]: functional ability assessed by the Health Assessment Questionnaire-Disability index (HAQ-DI); disease activity assessed by the Disease Activity Score in 28 joints (DAS28); joint count including tender joint count (TJC), swollen joint count (SJC) and Ritchie Articular Index (RAI), inflammatory markers including CRP and ESR; pain measured with the visual analogue scale (VAS) or the Short Form of the McGill Pain Questionnaire; and aerobic capacity assessed by VO_2_max and the Sit to Stand (STS) test.

### Data analysis and synthesis

Tables and forest plots were produced to tabulate and visually display the results of the individual studies and syntheses. RevMan5.3 software was used for statistical analysis. Since the measure of dispersion for change was not always available, we imputed a change-from-baseline standard deviation (SD) using a correlation coefficient as recommended by the Cochrane Handbook [31]. The measurement data used the mean difference (MD) or standard mean difference (SMD) as the effect analysis statistics, and each effect size provided a 95% CI. The heterogeneity among the results of the included studies was analysed by χ^2^ tests (test level at α = 0.1), and the degree of heterogeneity was quantitatively judged by combining with I^2^. Given I^2^ < 50% and *p* > 0.1, a fixed-effects model was used for analysis; if I^2^ > 50% and *p* < 0.1, a random-effects model was applied. The level of meta-analysis was set to α = 0.05. In addition, subgroup analysis was adopted. Two subgroups, short-term (≤ 16 w) and long-term (> 16 w), were divided according to the length of the intervention.

### Study quality assessments

The included studies’ risk of bias was evaluated independently and cross-checked by two investigators. The quality of the selected studies was scored using the quality critical appraisal criteria for RCTs, which is recommended by the Cochrane Handbook for Systematic Reviews of Interventions [31]. According to the Handbook, each item is rated as “low risk of bias”, “unclear risk of bias”, or “high risk of bias”.

### Sensitivity analysis

Sensitivity analysis was conducted to evaluate the robustness of the meta-analysis by assessing the influence of an individual study on the overall MD. We examined the effect of removing each study individually from the meta-analysis.

### Evidence quality assessment

The overall quality of the evidence for each outcome was rated by two reviewers using the Grading of Recommendations Assessment, Development, and Evaluation (GRADE) criteria [32]. In this system, the quality of the evidence is rated at one of four levels: high quality, moderate quality, low quality, and very low quality.

## Results

### Study selection

**Fig. 1:**
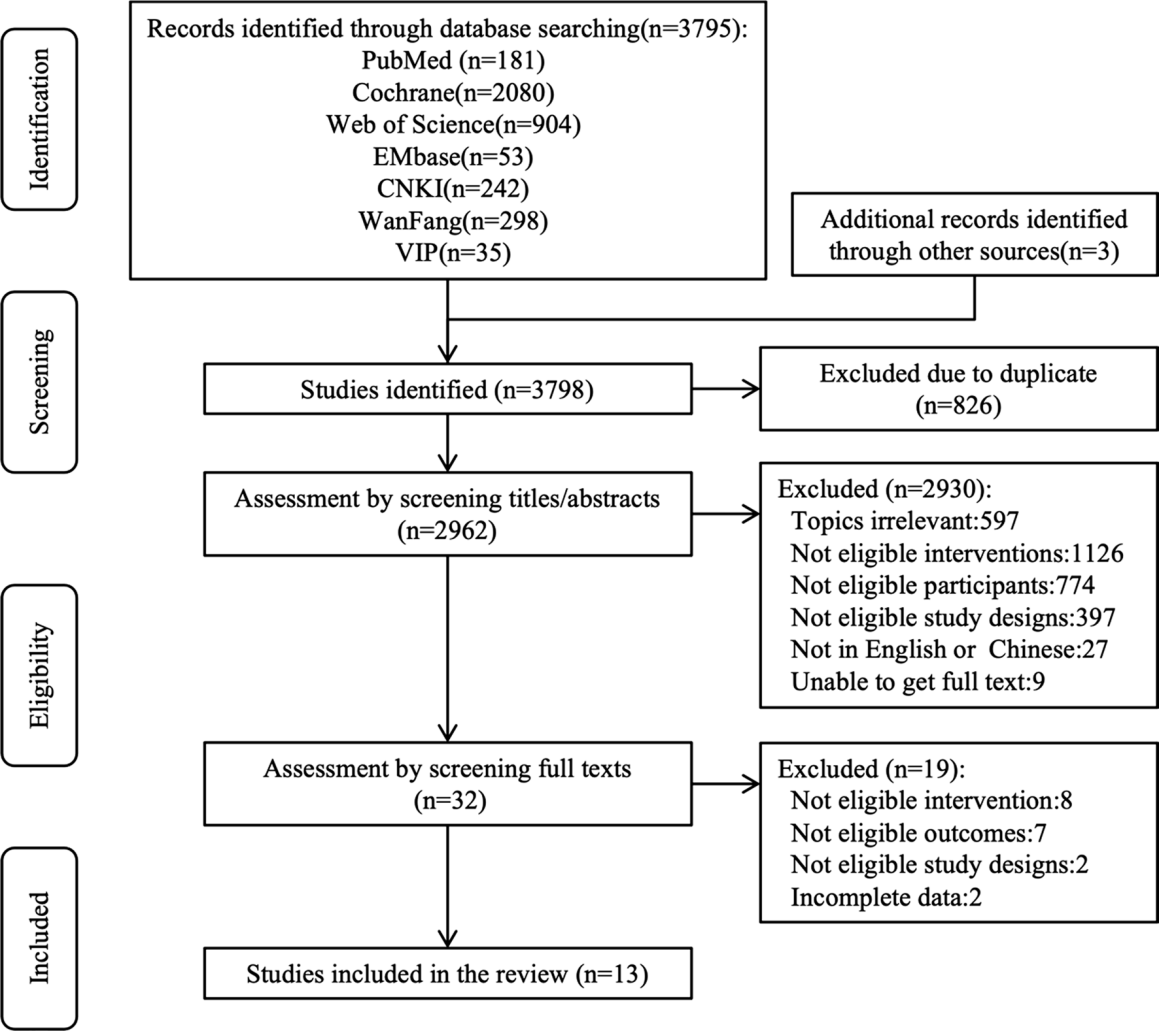
Study flow diagram

A total of 3798 articles were retrieved from 7 databases and other sources, among which 2962 remained after duplicates were removed. By browsing the title and abstract, 2930 articles were screened out for inappropriate intervention measures, study types, etc. The remaining 32 were assessed in full text independently against the eligibility criteria by two researchers. Nineteen articles were excluded: 8 articles’ intervention was not aerobic exercise; 7 articles’ outcome indicators did not meet the inclusion criteria; 2 articles’ study type did not match; and 2 articles had incomplete data results. Thirteen RCTs were finally included [33–45], studying a total of 967 RA patients. The study flow diagram for the systematic search process is shown in Fig. 1.

### Characteristics of the included studies

Table 1 summarizes the characteristics of the included studies. Thirteen trials were published between 2000 and 2020, and all of them were published in English. All patients were diagnosed with RA, accompanied by different degrees of functional status, and the average age ranged from 45 to 70.11. Aerobic exercise interventions included bicycle riding, running on a treadmill, jogging, and walking. The control group underwent a range of usual care in 7 studies [33, 34, 36, 38, 39, 41, 44], motion exercises in 1 study [35], education programs in 3 studies [37, 40, 45] and home-based nonaerobic exercises in 2 studies [42, 43]. (Table 1).

**Table 1:** Characteristics of the included studies

Author, year	Country	Function status	Sample (I/C)	Age (I/C)	Disease duration (I/C)	Intervention	Frequency (per week)	Duration (min)	Intensity	Length (week)	Comparison	Outcomes
Van den Ende, 2000	Netherlands	nr	34/30	62 ± 13/58 ± 14	8 ± 8/7 ± 8	Muscle strengthening exercises, bicycle training	3	15	60% of max HR	4	Usual care	①②④⑥⑦
De Jong, 2003	Netherlands	ACR I–III	150/150	54.0 ± 16/53.3 ± 18	7.5 ± 10.8/5 ± 7	Bicycle training, exercise circuit, sport or game	2	75	70–90% of max HR	96	Usual care	①②
Melikoglu,2006	Turkey	ACR I–II	20/20	46.42 ± 8.34/50.29 ± 9.70	6.72 ± 6.31/6 ± 5.67	Dynamic exercise performed on treadmill	2	20	60% of max HR	2	Range of motion (ROM) exercise	①⑤⑥⑦⑪
Neuberger, 2007	US	nr	84/75	55.5 ± 7.5	8 ± 12.38	Low-impact aerobics, strengthening	3	60	60–80% of max HR	12	Usual care	③⑤⑥⑧⑨
Baillet, 2009	France	ACR I–II	25/23	51.6 ± 8.3/56.3 ± 12.8	10.5 ± 8.0/11.7 ± 6.2	Cycling, running or resisting pulley cord,	5	45	60–80% of max HR	48	Education	①②
Flint-Wagner, 2009	US	ACR I–II	16/8	52.2 ± 13/49.0 ± 12.6	15.4 ± 10.8/11.2 ± 8.9	Strength training, aerobic exercise, abdominal exercises	3	75	nr	16	Usual care	①⑦
Breedland, 2011	Netherlands	Steinbrocker I–II	19/15	45 ± 11.9/51.8 ± 9.4	9.7 ± 14.0/5.9 ± 7.2	Muscle exercise circuit and bicycle training, sports, aqua jogging	2	90	More than 60% of max HR	8	Usual care	②⑨
Stavropoulos-Kalinoqlou, 2013	UK	nr	20/20	55.0 ± 9.8/52.8 ± 10.1	5.5 ± 1.68/7.0 ± 1.25	Treadmills, and cycle, hand and rowing erg-ometers	3	50–60	70% VO2 max	24	Education	①②⑤
Alghadir, 2016	Saudi Arabia	nr	20/20	45.9 ± 6.3/55.6 ± 12.41	12.3 ± 2.01/12.9 ± 3.01	Supervised aerobic exercise using treadmills	3	45–60	Moderate intensity, 30–45% of VO2max	24	Usual care	①②③④⑤⑥
Lange, 2019	Sweden	DAS28 < 5.1	36/38	69.14 ± 2.61/70.11 ± 2.30	15.4 ± 10.7/17.4 ± 10.9	Aerobic exercise and resistance exercis	3	30	70–89%of max HR	20	Home-based non-aerobic exercise	①⑨⑩
Andersson, 2020	Sweden	DAS28 < 5.1	24/25	69 ± 2.7/70 ± 2.4	13 ± 2.43/20 ± 3.25	Gym-based resistance and aerobic exercise	3	30	70–90%of max HR	20	Home-based non-aerobic exercise	①⑨⑩
Azeez, 2020	Ireland	nr	28/24	58.5 ± 9.75/63 ± 9.5	2 ± 4.75/9 ± 11.5	Cardiovascular exercise like walking, cycling or swimming and strength training	nr	nr	nr	12	Usual care	①②⑤⑥⑨
García-Morales, 2020	Mexico	ACR I–III	37/31	49.7 ± 11.4/49.1 ± 12.1	14 ± 3.88/8 ± 5	Articular rotation, bicycle, anaerobic exercise, recreational games,	2	80–90	65–85% of max HR	12	Education	①

① *HAQ-DI* Health Assessment Questionnaire-Disability Index, ② *DAS-28* Disease Activity Score 28, ③ *TJC* Tender joint count, ④ *SJC* Swollen joint count, ⑤ *CPR* C-reactive protein, ⑥ *ESR* erythrocyte sedimentation rate, ⑦ *VAS* visual analogue scale, ⑧ McGill Pain Questionnaire, ⑨*VO2max* maximal oxygen consumption, ⑩ *STS test* Sit to Stand test, ⑪ *RAI* Ritchie articular index, *nr* not reported/inapplicable, *HR* heart rate, *ACR* American College of Rheumatology, *DEP* dynamic exercise program

### Quality appraisal

**Fig. 2:**
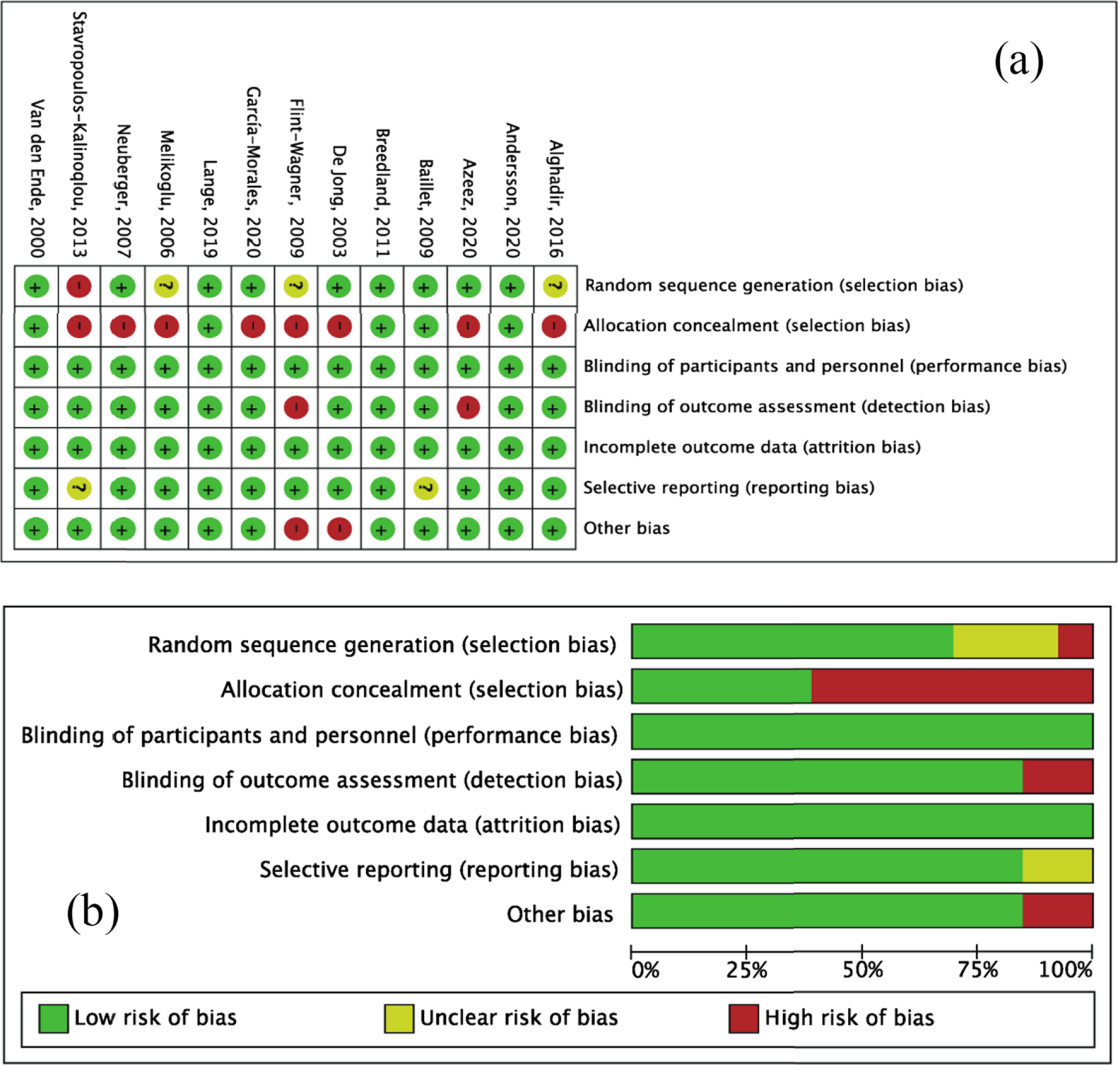
Risk of bias. **a** Risk of bias summary and **b** risk of bias graph

The risk of bias is shown in Fig. 2. Three trials [35, 38, 41] (23.08%) had inadequate random sequence generation due to only stating that they were randomized without describing a specific random method. One trial [40] (7.69%) lacked a randomized design. Five trials [33, 37, 39, 42, 43] (38.46%) had allocation concealment by using sealed and opaque envelopes or sealed tickets, and the other trials did not report the use of allocation concealment. None of the trials reported the blinding of participants and personnel. However, in trials of aerobic exercise interventions, it was difficult to blind the health-care provider or participant; moreover, the primary outcomes mainly were objective indicators that were hardly affected by blinding, so we regarded this aspect as low risk. Two trials [38, 44] (15.38%) were rated as high risk for not reporting assessor blinding. Two trials [37, 40] (15.38%) were rated as unclear risk for not reporting a partial outcome indicator. Two other trials [34, 38] (15.38%) were rated as at other high-risk bias based on the unbalanced sample size between two groups and slight differences between the groups at baseline, respectively (Fig. 2).

### Assessment of efficacy

For the included studies, we performed a meta-analysis of 7 outcomes: functional ability, disease activity, joint counts, inflammatory markers, pain, aerobic capacity and STS test. The following is a summary of the data evaluated for each outcome in each field.

#### Functional ability

##### HAQ-DI

**Fig. 3:**
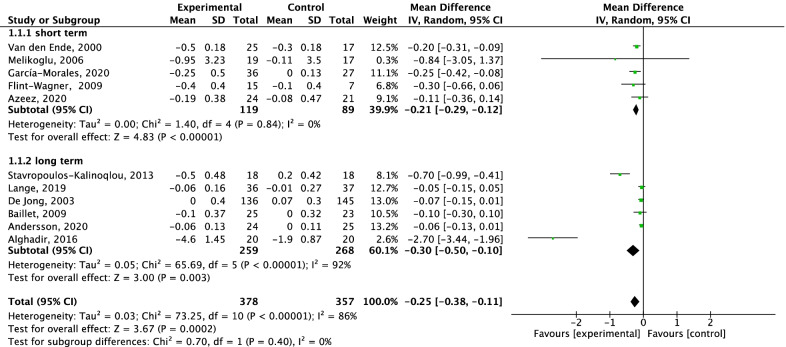
Forest plot: influence of aerobic exercise interventions on HAQ-DI score change

A total of 11 RCTs [33–35, 37, 38, 40–45] reported changes in HAQ-DI scores, including 735 patients (378 patients in the experimental group and 357 patients in the control group). The random-effects model analysis results showed that the total change in the intervention group was significantly lower than that in the control group [MD = − 0.25, 95% CI (− 0.38, − 0.11), *P* = 0.0002]. No significant difference was found between long-term and short-term aerobic exercise (*P* = 0.40) (Fig. 3).

#### Disease activity

DAS-28.

##### Joint count

**Fig. 4:**
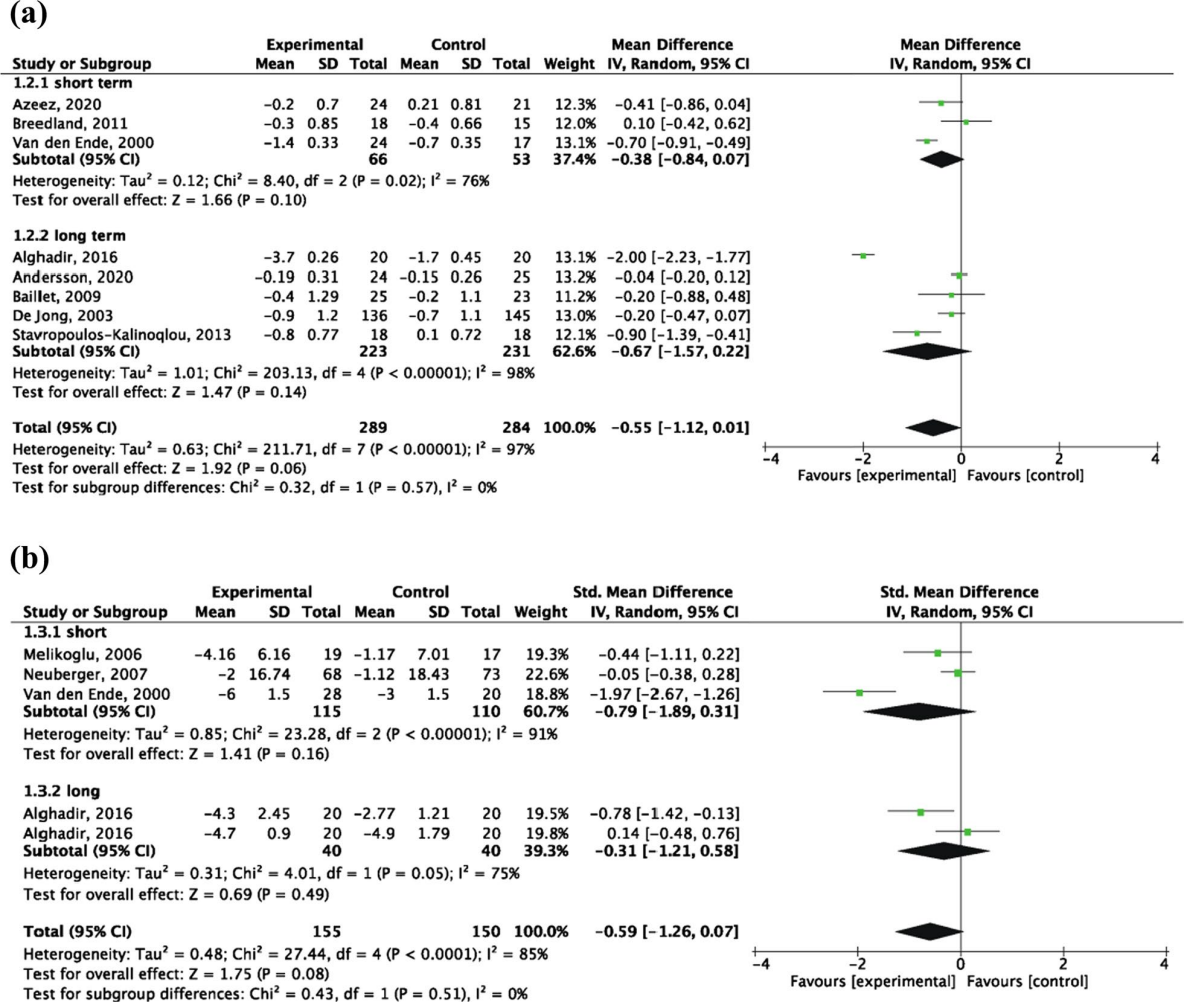
Forest plot: **a** influence of aerobic exercise interventions on DAS-28 score change; **b** influence of aerobic exercise interventions on joint count change

Disease activity, assessed by the DAS-28, was recorded in 8 RCTs [33, 34, 37, 39–41, 43, 44], including 573 patients (289 patients in the intervention group and 284 patients in the control group). The random-effects model analysis results showed that the DAS-28 score change of the intervention group was lower than that of the control group, but the difference was not statistically significant [MD = − 0.55, 95% CI (− 1.12, 0.01), *P* = 0.06]. No significant difference was found between long-term and short-term aerobic exercise (*P* = 0.57) (Fig. 4a).

A total of 4 RCTs [33, 35, 36, 41] were included, of 305 patients (155 patients in the experimental group and 150 patients in the control group). RAI was reported in 1 trial [35], SJC in 2 studies [33, 41] and TJC in 2 studies [36, 41]. The random-effects model analysis results showed that the joint count change of the intervention group was lower than that of the control group, but the difference was not statistically significant [SMD = − 0.59, 95% CI (− 1.26, 0.07), *P* = 0.08]. No significant difference was found between long-term and short-term aerobic exercise (*P* = 0.51) (Fig. 4b).

#### Inflammatory markers

##### CRP

**Fig. 5:**
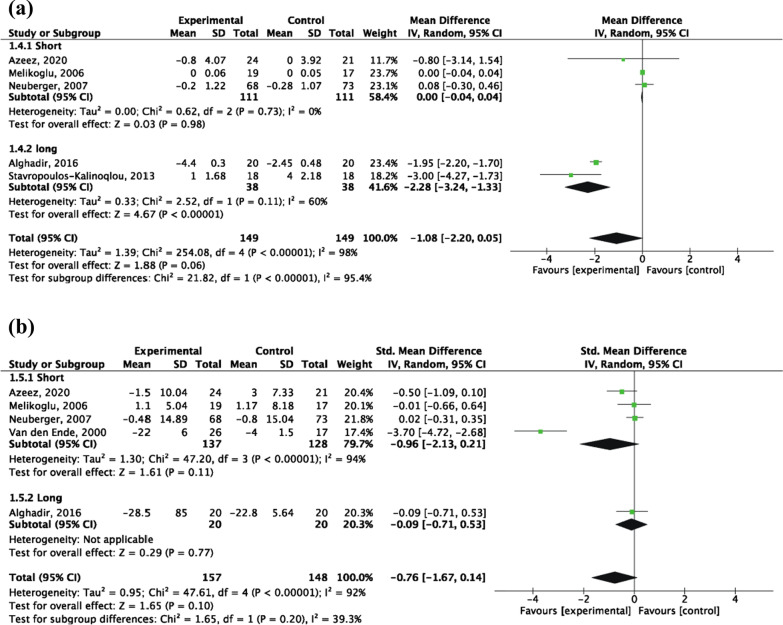
Forest plot of Inflammatory markers: **a** influence of aerobic exercise interventions on CRP change; **b** influence of aerobic exercise interventions on ESR change

A total of 5 RCTs [35, 36, 40, 41, 43] were included, of 298 patients (149 patients in the experimental group and 149 patients in the control group). Random-effects model analysis results showed that the CRP change of the intervention group was lower than that of the control group, but the difference was not statistically significant [MD = − 1.08, 95% CI (− 2.20, 0.05), *P* = 0.06]. However, the subgroup analysis showed that long-term aerobic exercise significantly decreased CRP levels [MD = − 2.28, 95% CI (− 3.24, − 1.33), *P* < 0.00001], but not in the short term (Fig. 5a).

##### ESR

A total of 5 RCTs [33, 35, 36, 41, 44] were included, of 305 patients (157 patients in the intervention group and 148 patients in the control group). The random-effects model analysis results showed that the ESR change of the intervention group was lower than that of the control group, but the difference was not statistically significant [SMD = − 0.76, 95% CI (− 1.67, 0.14), *P* = 0.10]. No significant difference was found between long-term and short-term aerobic exercise (*P* = 0.20) (Fig. 5b).

#### Pain

**Fig. 6:**
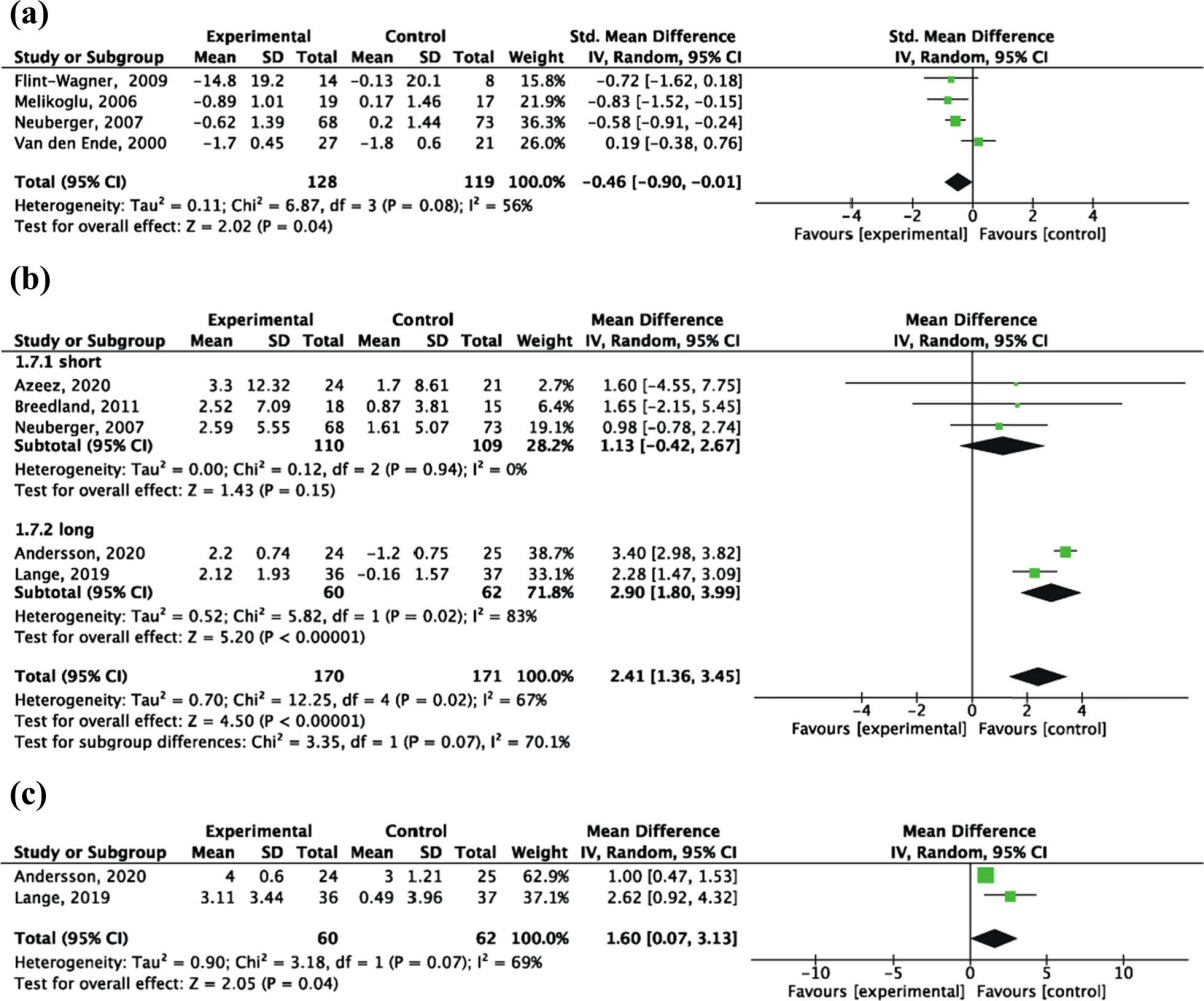
Forest plot of Inflammatory markers: **a** influence of aerobic exercise interventions on pain change; **b** influence of aerobic exercise interventions on VO_2_max change; **c** influence of aerobic exercise interventions on STS score change

A total of 4 RCTs [33, 35, 36, 38] of short-term interventions were included, of 247 patients (128 patients in the experimental group and 119 patients in the control group). The VAS was reported in 3 trials [33, 35, 38], and the McGill Pain Questionnaire was reported in 1 study [36]. Random-effects model analysis results showed that the pain change of the intervention group was significantly lower than that of the control group [SMD = − 0.46, 95% CI (− 0.90, − 0.01), *P* = 0.04] (Fig. 6a).

#### Aerobic capacity

##### VO_2_max

A total of 5 RCTs [36, 37, 42–44] were included, of 247 patients (170 patients in the experimental group and 171 patients in the control group). Random-effects model analysis results showed that the VO_2_max change of the intervention group was significantly higher than thecontrol group [MD = 2.41, 95% CI (1.36, 3.45), *P* < 0.00001] In addition, compared to short-term aerobic exercise, long-term aerobic exercise had a better potential improvement trend, although at a significant difference was not shown (*P* = 0.07) (Fig. 6b).

#### Sit to Stand test

Two RCTs [42, 43] of long-term interventions were included, of 122 patients (60 patients in the experimental group and 62 patients in the control group). Random-effects model analysis results showed that the STS score change of the intervention group was significantly higher than that of the control group [MD = 1.60, 95% CI (0.07, 3.13), *P* = 0.04] (Fig. 6c).

### Adverse events

Six studies [34–38, 40] reported the safety of the experiment, and all stated that no adverse effects or detrimental disease status effects were found. The pooled results of the 13 RCTs indicate that aerobic exercise exerts no severe side effects and is generally safe for humans with RA.

### Sensitivity analysis

The selected studies were removed one by one, and the overall effect results were recalculated. The overall effect results of functional ability, disease activity, inflammatory markers, joint count and aerobic capacity were relatively stable. After the study of Van den Ende [33] was eliminated, the results of the overall effect of pain score changed (I^2^ = 0%, SMD = − 0.64, 95% CI [− 0.92, − 0.35], *P* < 0.0001), indicating that the study was a source of heterogeneity and was strongly affecting the results. After removing the study of Andersson [43], the overall effect of aerobic capacity did not change significantly, but the heterogeneity was reduced (I^2^ = 0%, MD = 2.03, 95% CI [1.32, 2.75], *P* < 0.00001), suggesting that this study was the source of heterogeneity in the aerobic capacity database.

### GRADE evidence assessment

GRADEpro was used to evaluate the evidence. In terms of the limitations of the studies, the blinding and allocation concealment reported in some included studies were insufficient, but it had little effect on the experimental results, and they were not downgraded. In terms of inconsistency, most of the indicators were statistically heterogeneous, and they were all downgraded. In terms of indirectness, there was no significant difference between PICO and the research aim. In terms of inaccuracies, some of the outcome sample sizes did not meet the optimal sample size, and some of the outcome effect sizes crossed the invalid line. In terms of publication bias, the included literature does not involve commercial interests, and the literature was searched comprehensively. Currently, no clear evidence has shown a risk of bias, and the grade has not been downgraded. See Table 2 for details.

**Table 2:** Evidence assessment of outcomes

Outcomes	Anticipated absolute effects (95% CI)	Patients (studies)	Quality of evidence	Rated down reasons
Function ability	0.25 MD lower (0.38–0.11 lower)	735 (11 RCTs)	⨁⨁⨁◯ Moderate	Inconsistency
Disease activity	0.55 MD lower (1.12 lower to 0.01 higher)	573 (8 RCTs)	⨁⨁◯◯ Low	Inconsistency; imprecision
Joint counts	0.59 MD lower (1.26 lower to 0.07 higher)	305 (4 RCTs)	⨁⨁◯◯ Low	Inconsistency; imprecision
CRP	1.08 MD lower (2.20 lower to 0.05 higher)	298 (5 RCTs)	⨁⨁◯◯ Low	Inconsistency; imprecision
ESR	0.76 SMD lower (1.67 lower to 0.14 higher)	305 (5 RCTs)	⨁⨁◯◯ Low	Inconsistency; imprecision
pain	0.46 SMD lower (0.90–0.01 lower)	247 (4 RCTs)	⨁⨁⨁◯ Moderate	Imprecision
Aerobic capacity	2.41 MD higher (1.36–3.45 higher)	247 (5 RCTs)	⨁⨁◯◯ Low	Inconsistency; imprecision
STS test	1.60 MD higher (0.07–3.13 higher)	122 (2 RCTs)	⨁⨁◯◯ Low	Inconsistency; imprecision

## Discussion

In general, this systematic review and meta-analysis demonstrated that aerobic exercise could significantly partially ameliorate important RA patient outcomes: functional ability, pain, and aerobic capacity. There was a trend towards a greater reduction in the outcomes of disease activity, joint count, and inflammatory markers, but the differences were not statistically significant in either group. No adverse events were reported, indicating that aerobic exercise is safe for RA patients.

RA is often accompanied by poor physical function. The Health Assessment Questionnaire disability index (HAQ-DI) is a popular index for measuring the function of joints by the patients’ multidimensional ability to perform activities of daily living [37]. With good reliability and validity, the HAQ-DI helps to detect functional changes, monitor the severity of disease, and predict all‐cause mortality in RA patients [46–48]. This study’s analysis result shows that aerobic exercise can effectively reduce the HAQ-DI score, which is consistent with the analysis result of Bailet [49], indicating that aerobic exercise is of great significance for improving functional ability.

Another major symptom of RA is pain, which classically occurs in the small joints of the hands, wrists and feet [50]. Despite the successful clinical application of medicine, pain persists for many RA patients. Among the 4 studies reporting the effects of aerobic exercise on the pain of RA patients, 3 used the VAS scale,1 used the McGill Pain Questionnaire, and all were short-term interventions (≤ 16 weeks). The results showed that aerobic exercise has a pain-relieving effect, and a similar reduction in pain has been found in previous studies [49, 51]. An effect of aerobic exercise on pain relief has been found for chronic low back pain [52], knee osteoarthritis [53] and other conditions. The mechanism may be related to the modification and influence of exercise on central pain processing and pain sensitivity [54].

Aerobic capacity is reflected in the value of VO_2_max, a strong and independent predictor of overall mortality, and it is associated with disease activity and cardiovascular risk factors in early rheumatoid arthritis [44, 55]. This research found improved VO_2_max levels after aerobic exercise intervention; similarly, an absolute improvement in cardiorespiratory fitness in RA patients was shown after a 10-week high-intensity interval walk training programme [56]. The results demonstrated that exercise could improve aerobic capacity and reduce the risk of CVD and mortality in RA patients.

The advantage of aerobic exercise is also reflected in the improvement of the STS score, which is used to evaluate muscle strength of the lower limbs, mobility and risk of falls [57]. Aerobic exercise may increase body mobility, thereby reducing the impairment of functional status in RA patients. In addition, functional ability represents the main factor related to high fall prevalence in RA patients [58], suggesting that STS and HAQ can together be listed as the main outcomes for discussion in future studies.

The three indicators reflecting disease activity, including DAS-28, joint counts and inflammatory markers, were not significantly improved compared with the control group. This result is consistent with previous studies [49, 59]. Large heterogeneity was seen in the results of three outcomes, indicating systematic differences among the included studies, resulting in the reliability of the overall results being less stable. Therefore, the significance of the included studies concerning the change in DAS-28, joint counts and inflammatory markers cannot be reliably interpreted. Notably, subgroup analysis showed that long-term (> 16 weeks) aerobic exercise intervention resulted in a significant reduction in CRP. Subgroup analysis indicated that long-term interventions tended to be more effective than short-term exercise in terms of functional ability, disease activity, inflammatory markers and aerobic capacity indicators. This shows a trend towards better improvement with long-term exercise for RA patients. However, due to the small number of included articles and samples, more evidence is needed in the future to validate these findings.

A well-balanced duration, frequency and length of training is essential for RA patients. The ACSM recommends that most adults engage in moderate-intensity cardiorespiratory exercise training for ≥ 30 min·day on ≥ 5 days·week for a total of ≥ 150 min·week [60]. The included articles did not focus on clearly demonstrating the impact of exercise time and frequency on the results. Additional studies are needed in the future to explore the dose response relationship between exercise intensity and RA symptoms.

Through GRADE classification, this study found varying quality of evidence to show the effects of aerobic exercise on RA patient outcomes. In terms of functional ability, moderate-quality evidence indicated that aerobic exercise could reduce HAQ scores. Low-quality evidence indicated that aerobic exercise reduced the DAS-28 score and joint counts and decreased inflammatory markers without a significant effect. Moderate-quality evidence suggests that aerobic exercise can alleviate RA patients’ pain. Low-quality evidence showed that aerobic exercise significantly increased the VO2max and STST scores of RA patients.

Compared with previously published reviews, this meta-analysis was more focused on aerobic patterns, incorporated more new studies, summarized the latest evidence of the effect of aerobic exercise programs in RA, and integrated more outcome indicators than have been analysed before, such as STS score and VO_2_max levels [17, 25, 61, 62]. Meanwhile, this study has certain limitations. First, due to the limitations of the included literature, it does not include indicators such as quality of life, emotional health, fatigue, etc. Second, part of the data included in the combined analysis study was obtained after conversion, and there may be slight deviations from the original data. Some indicators appeared, only in two studies. Moreover, the methodological quality of the included studies was generally low to moderate, calling for more extensive and higher-quality studies in the future. The advantage of this review is its up-to-date, systematic and comprehensive literature search on related topics and innovative attention to two new indicators: aerobic capacity and STS.

## Conclusion

Current research shows that aerobic exercise has a beneficial effect on the functional capacity, pain and aerobic exercise capacity of rheumatoid arthritis patients and exerts no adverse events. However, there is no substantial evidence to prove that aerobic exercise significantly affects disease activity, joint count, and inflammatory markers in RA patients. More RCTs with larger sample sizes and higher quality are warranted.

## Data Availability

The PubMed, Cochrane Library, Web of Science, Embase, CNKI, WanFang Data and VIP databases were searched for eligible articles. Additionally, this study was registered with the PROSPERO database (registration number: CRD42021242953). Full data for this research is available through the corresponding author upon request.

## Abbreviations

RA: Rheumatoid arthritis
NSAIDs: Non-steroidal anti-inflammatory drugs
DMARDs: Disease-modifying antirheumatic drugs
VO2max: Peak oxygen consumption
RCTs: Randomized controlled trials
HAQ-DI: The Health Assessment Questionnaire-Disability index
DAS28: The Disease Activity Score in 28 joints
TJC: Tender joint count
SJC: Swollen joint count
RAI: The Ritchie Articular Index
CRP: C-reactive protein
ESR: Erythrocyte sedimentation rate
VAS: Pain on visual analog scale
STS: Sit to Stand test
GRADE: The Grading of Recommendations Assessment, Development, and Evaluation
